# A model of the organizational resilience of hospitals in emergencies and disasters

**DOI:** 10.1186/s12873-024-01026-6

**Published:** 2024-06-24

**Authors:** Fatemeh Seyghalani Talab, Bahman Ahadinezhad, Omid Khosravizadeh, Mohammad Amerzadeh

**Affiliations:** 1 Student Research Committee, Department of Healthcare Management, School of Public Health, Qazvin University of Medical Sciences, Qazvin, Iran; 2 Social Determinants of Health Research Center, Research Institute for Prevention of Non-Communicable Diseases, Qazvin University of Medical Sciences, Qazvin, Iran

**Keywords:** Model, Resilience, Hospital, Disaster, Emergency

## Abstract

**Background:**

In the health system, hospitals are intricate establishments that offer vital medical services. Their resilience plays a crucial role in mitigating the societal repercussions of disasters. A hospital must possess the capacity to withstand risks, preserve its fundamental structure and operations, and enhance its preparedness by augmenting various capabilities and promptly recovering from the impacts of potential risks. It enables the hospital to attain a heightened level of readiness. Therefore, this study aimed to develop a resilience model tailored for hospitals to navigate crises and disasters effectively.

**Methods:**

This mixed-method study was conducted in 2023 in three phases: (1) Identification of the factors influencing the organizational resilience of the hospital, (2) Evaluation of the influential factors by an expert panel. (3) Following the standardization process, we administered 371 questionnaires to individuals, such as university staff managers and supervisors, nursing managers, and research unit managers. The sample size was determined by multiplying the components by 10, resulting in 360 (10 * 36). Therefore, we selected a sample size of 371 participants. Structural Equation Modeling (SEM) was employed to examine the causal relationships between variables. These steps were performed using SPSS 25.0 and AMOS 22 software. Finally, we identified and presented the final model. We utilized AMOS 22 and applied the SEM to assess the correlation between the variables, with a significance level of 0.05.

**Results:**

Findings indicate that the appropriate modeling identified five dimensions comprising 36 components. These dimensions include vulnerability, preparedness, support management, responsiveness and adaptability, and recovery after the disaster. The model demonstrates a good fit, as indicated by the X2/d indices with a value of 2.202, a goodness of fit index (GFI) of 0.832, a root mean square error of estimation (RMSEA) of 0.057, an adjusted comparative fit index (CFI) of 0.931, and a smoothed fit index (NFI) of 0.901.

**Conclusion:**

Enhancing hospital resilience is crucial for effective preparedness and response to accidents and disasters. Developing a localized tool for measuring resilience can help identify vulnerabilities, ensure service continuity, and inform rehabilitation programs. The proposed model is a suitable framework for assessing hospital resilience. Key factors include human resource scarcity, hospital specialization, and trauma center capacity. Hospitals should prioritize efficient resource allocation, information technology infrastructure, in-service training, waste management, and a proactive organizational framework to build resilience. By adopting this approach, hospitals can better respond to crises and disasters, ultimately reducing casualties and improving overall preparedness.

**Supplementary Information:**

The online version contains supplementary material available at 10.1186/s12873-024-01026-6.

## Background

Global impacts are caused by crises and disasters, encompassing a range of events, such as extreme weather phenomena, natural disasters, bioterrorism incidents, and outbreaks of infectious diseases [1]. Due to these crises and disasters, the affected communities endure diverse damages, injuries, and fatalities [2, 3]. Disasters of this nature can potentially disrupt a wide range of infrastructures and facilities, including hospitals, schools, transportation systems, and emergency services. During critical situations, hospitals can mitigate the impacts and minimize mortality rates associated with such circumstances [4]. In light of this, ensuring effective disaster management within hospitals becomes imperative to ensure the uninterrupted provision of healthcare services, even when hospitals are directly impacted by the one crucial component in emergency response and maintaining order during critical emergencies [5]. Having pre-established operational plans to enhance hospital preparedness during critical and emergency circumstances is critical to effectively responding to and managing emergencies [6, 7]. Creating resilience within the health system is a crucial stage in health disaster management, as it enables the continued provision of healthcare services [8]. Hospital resilience is the capacity of hospitals to withstand, assimilate, and respond to the impacts of critical situations, all while ensuring the uninterrupted delivery of essential healthcare services [9].

Hospital resilience includes returning to the initial state or adapting to new conditions. This definition encompasses a comprehensive perspective on the hospital’s capacity to respond to emergencies effectively. It encompasses the hospital’s inherent strength to withstand and absorb crises and its adaptive flexibility in implementing strategies to ensure the uninterrupted provision of essential health services and adapt to future crises [10].

A resilient system can endure environmental pressures, enabling optimal performance even in disaster. Hospital resilience is closely linked to reducing vulnerability to shocks caused by natural disasters while simultaneously enhancing adaptive capacity through improved actions and opportunities [11, 12]. Hosseini defined resilience as the capacity and ability of an organization to absorb and tolerate adverse effects and quickly restore performance [13]. Several researchers have presented a framework for understanding resilience and proposed methods for measuring it within hospital settings [10, 14]. Another study identified resource capability, equipment, and organizational structure as factors that significantly impact hospital resilience [15]. Jalgenejad et al. highlighted dimensions such as management, staff, and hospital infrastructure as crucial factors contributing to hospital resilience [16]. Zaboli et al. classified the factors influencing resilience in military hospitals into five primary dimensions: hospital vulnerability and safety, disaster preparedness, capacity adaptation, continuity of services during crises, and rehabilitation and adaptation to post-disaster conditions [17]. Mohtadi Ali et al. introduced a decision support model to enhance hospitals’ resilience against disasters. This model encompasses preventive and systemic improvements, from forecasting to managing and monitoring the organization’s performance during disasters [18]. Recognizing the importance of measurement for effective control and management, hospitals need to establish specific standards aligned with the four phases of disaster management (prevention, preparation, coping, and recovery) [19]. To effectively manage and control their resilience, hospitals should implement a system that enables the measurement of resilience within these phases [8]. Evaluation and auditing are crucial indicators of improvement and progress in all organizations, regardless of whether they follow traditional or modern management approaches. The evaluation process typically involves qualitative assessment, with specific indicators being evaluated semi-quantitatively. The nature and quality of the evaluation can vary depending on the evaluators’ capabilities [8]. Enhancing resilience necessitates using standardized tools to identify, evaluate, and prioritize the components of resilience. By implementing control measures, these tools aid in preventing adverse events and minimizing their negative consequences [20, 21]. Given the potential for irrevocable losses and damages resulting from disruptions and incompatibilities within hospitals, there is a pressing need to motivate researchers and decision-makers to develop solutions to enhance resilience in healthcare [22].

Therefore, given the significance of uninterrupted service delivery in hospitals and the criticality of evaluation, monitoring, and planning to enhance hospitals’ resilience against accidents, it is evident that the existing studies in this domain lack conclusive evidence. Therefore, further research is warranted to garner more comprehensive insights and address the existing gaps in understanding.

This study aimed to identify the influential dimensions impacting hospital centers’ organizational resilience and develop a model tailored for hospitals to navigate crises and disasters effectively.

## Methods

We conducted a descriptive-analytical research study using a mixed-method design in 2023. The study was structured into three distinct stages, as outlined below:

### Comprehensive literature review

The first stage of the research focused on evaluating the findings of relevant literature on the organizational resilience of educational hospitals. To ensure the reliability and validity of the content, minimize bias, and uphold integrity, we employed a data collection form as a tool during the research process. We searched the keywords “Sustainability”, “Resilience” ، “Strategy”, “Medical centers”, “Healthcare”, “Hospital”, “Organizational resilience”, “Crisis”, “Disaster”, “Emergency”, “Health services”, “Medical services”, and “Health system” in Google Scholar, PubMed, Scopus, Web of Science, Science Direct, MagIran, SID and Irandoc. The inclusion criteria were: (1) Studies carried out within the last decade in hospital organizational resilience; (2) All types of descriptive, analytical, and cross-sectional studies that utilize various methodologies; and (3) Studies written only in English and Persian languages. Meanwhile, the exclusion criteria were studies that were conducted in other areas and studies that were not accessible. Then, we evaluated the obtained articles qualitatively. Finally, we categorized the factors affecting the organizational resilience of the hospital and arranged the most important ones in one division. The comprehensive review has been previously published elsewhere [9].

### Expert panel

During the panel stage, we tried to recontextualize the factors influencing the resilience of medical centers derived from the comprehensive review stage by engaging with experts in the field. Based on the thematic background, experts identified the influential dimensions of resilience specific to Qazvin Medical Centers, Iran. The panel comprised individuals with ample knowledge and experience in various domains such as hospital services, health management, health policy-making, healthcare in disasters, nursing, and related fields. The research was conducted within the educational centers of Qazvin province. The panel members from each center comprised individuals such as president, manager, nursing manager, financial manager, public affairs manager, and hospital manager where resilience had been implemented or was planned to be implemented. Two expert panels were conducted under the management of the research team, and ten experts attended each meeting. The duration of each session was 120 to 180 min. In the final phase, experts compiled and categorized the themes and concepts derived from the review process.

**Fig. 1:**
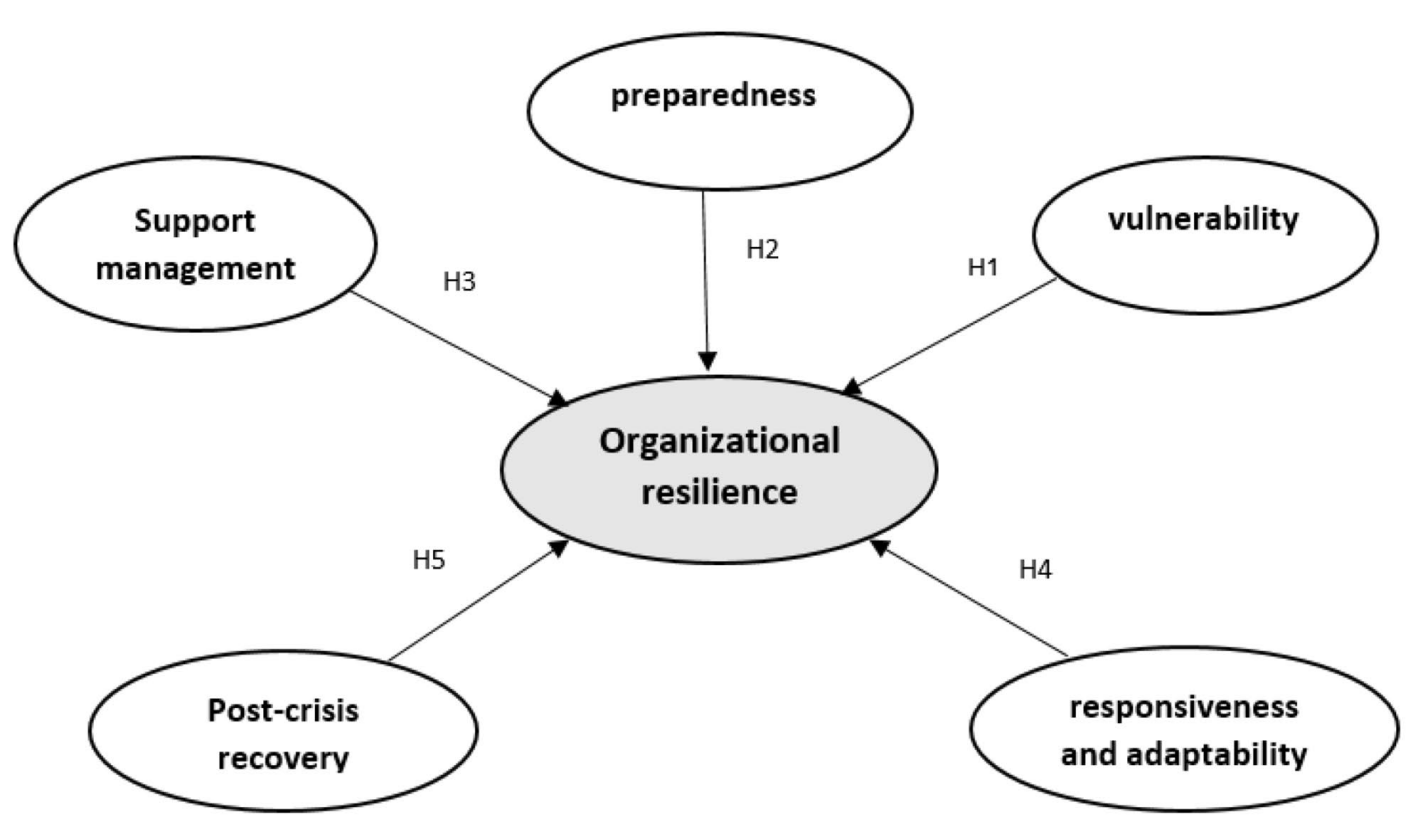
Conceptual Model of study

The resulting conceptual model of the study is depicted in Fig. 1. The development of this model is a culmination of an extensive analysis of existing research literature findings, as well as the conceptualization and classification carried out by an expert panel.

Based on the literature survey, we proposed the following hypotheses:

#### H1:

Vulnerability has a significant effect on organizational resilience.

#### H2:

Preparedness has a significant effect on organizational resilience.

#### H3:

Support management has a significant effect on organizational resilience.

#### H4:

Responsiveness and adaptability during a disaster significantly affect organizational resilience.

#### H5:

Post- disaster recovery has a significant effect on organizational resilience.

### Preliminary Model Development and Instrumentation

A questionnaire was created and made available to a panel of 10 expert professionals to evaluate the formal validity of the research instrument during this phase. The panel consisted of four individuals specializing in health system management, three experts in policy-making and health during accidents and disasters, and three experienced doctors and clinical staff members. Upon reviewing the intended dimensions and components, they verified the face validity of the questionnaire. Subsequently, the content validity of each question in the questionnaire was assessed by calculating the CVR index. The findings indicated that all the questions in the questionnaire demonstrated satisfactory content validity, as evidenced by their CVR values exceeding the threshold of 0.64. Cronbach’s alpha coefficient was employed to assess the reliability of the questionnaire, yielding a total value of 0.943. This indicates that the developed questionnaire possesses acceptable reliability.

The developed questionnaire consisted of demographic information and questions about the perceived importance of conceptualized dimensions and components. A five-point Likert scale was utilized for respondents to rate their responses. The initial section of the questionnaire comprised five inquiries about age, gender, level of education, length of service, and organizational position. In the second section of the questionnaire, 36 questions were formulated and categorized into five distinct dimensions. The significance of factors influencing the organizational resilience of hospitals was analyzed using a five-point Likert scale, ranging from “very low” to “very high.” The statistical population comprised university managers, chiefs, nursing managers, and research unit managers from Quds, Rajaee, Velayat, Bu Ali, Kosar, and 22 Bahman hospitals. The sample size was determined by multiplying the components by 10, resulting in 360 (10 * 36). Therefore, we selected a sample size of 371 participants. This sampling method followed the rationale proposed by James Stevens, which involves selecting 5 to 15 samples per component. This approach is well-suited for conducting multiple regression analysis using techniques such as standard least squares And Structural Equation Modeling (SEM) models [23]. Only fully completed questionnaires were included in the analysis, and incomplete questionnaires were excluded. Descriptive statistics were computed using the SPSS 25 software.

### Validation and presentation of the final model

**Fig. 2:**
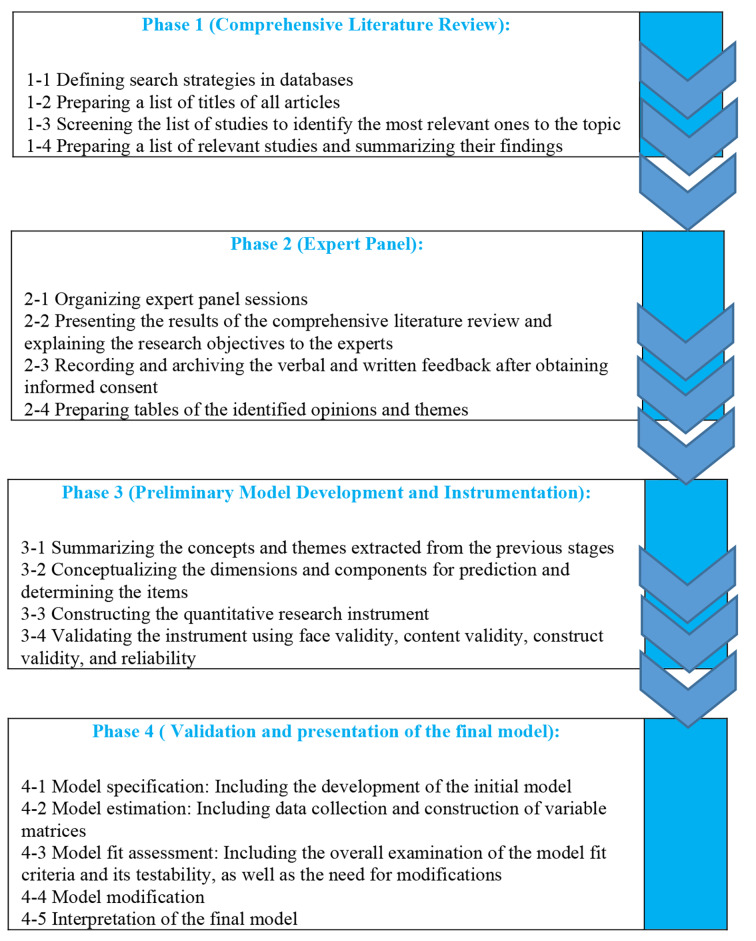
Demonstrates the methodology

A final model was developed during this phase to analyze the quantitative results. The SEM was employed to examine the causal relationships between variables in a unified framework and to present the final model. This approach consists of five steps: model specification (initial model structure), model estimation (data collection and formulation of the variable matrix), model fit assessment (comprehensive evaluation of the model’s adequacy, feasibility, and identification of necessary adjustments), model refinement, and interpretation of the results. Additionally, we employed five indices to evaluate the fitness of the SEM and CFA models. The χ2 index was used to assess the overall fitness of the model and determine any discrepancies between the observed and estimated covariance matrices. The approximate variances and covariances were evaluated throughout the model using the goodness-of-fit index (GFI). The root mean square error of approximation (RMSEA) was used to assess the mean discrepancy between the model’s covariance matrix and the data’s covariance matrix. We compared the proposed model with an independent model using the normed fit index (NFI) and the comparative fit index (CFI) within the context of confirmatory factor analysis (CFA). We assessed the Chi-square values of the independent model using the NFI. These steps were performed using SPSS 25.0 and AMOS 22 software. Finally, we identified and presented the final valid model. Fig. 2 demonstrates the methodology.

## Results

### Review dimensions and components

Multiple studies were conducted at different stages of the research. From the database search, 2442 studies were identified, of which 731 articles were rejected due to duplication. After screening the titles and abstracts, 1619 more studies were excluded; then, 62 articles were excluded from the study process after full-text screening, and finally, 23 articles were selected that were fully compatible with the study objectives. In the literature review, a comprehensive search was conducted to identify relevant studies on hospital organizational resilience. The search process followed the PRISMA guidelines. Initially, 2,442 studies were identified from database searches. After removing 731 duplicate records, 1,711 studies underwent title and abstract screening, and 1,619 were excluded based on the predefined exclusion criteria. These criteria included studies that were not focused on hospitals, did not assess organizational resilience, or were not empirical research articles. The remaining 92 full-text articles were assessed for eligibility, and 62 were excluded. The primary reasons for exclusion at this stage were: (1) the study did not specifically examine factors influencing hospital organizational resilience, (2) the study population was not healthcare-related, or (3) the study design was not appropriate (e.g., case reports, commentaries). Finally, 23 studies were selected for inclusion in the literature review, as they were fully aligned with the study’s objectives. After the initial search, the researchers evaluated the articles using the Strengthening the Reporting of Observational Studies in Epidemiology (STROBE) checklist to assess their quality. This checklist is a widely recognized tool for evaluating the quality of observational studies, as it encompasses 22 criteria across sections such as title, introduction, methodology, results, discussion, and conclusion. Only articles that met over 50% of the checklist criteria were selected for further consideration.

Following the quality assessment, the researchers proceeded with data extraction. This process involved systematically extracting and summarizing key information from the included articles, such as the author, year of publication, study location, title, target population, study type, methodology, and the main findings of each study. By employing a rigorous quality assessment process using the STROBE checklist and extracting relevant data, the researchers ensured that the included studies met a certain standard of quality and that the necessary information was captured for the subsequent stages of the review.

### Participants

The average age of the participants was 33.54 years. The highest number of participants was in the age group of 20 to 30 years, and the lowest number of participants was over 50 years old. Besides, 96 people (25.9%) were men and 275 were women (74.1%). Most of the participants had a bachelor’s degree (64.4%). A specialized doctorate (4.9%) had the lowest frequency. Regarding the service experience of the participants, the highest number (*n* = 205, 55.3%) had less than ten years of experience, and the lowest number (*n* = 52, 14.0%) had more than twenty years of experience. Regarding organizational positions, 290 participants (78.2%) were employed in hygiene and clinical positions (Table 1).

**Table 1:** Demographics distribution of participants

Variables	Components	Frequency	Percentage
Age	Less than 30	168	45/3
	31–40	122	32/9
	41–50	70	18/9
	More than 50	11	3/0
Sex	Female	275	74/1
	Male	96	25/9
Level of education	Diploma or less	16	4/3
	Bachelor	239	64/4
	MA	79	21/3
	PhD	18	4/9
	MD	19	5/1
Service experience	Less than 10 years	205	55/3
	10–20 years	114	30/7
	More than 20 years	52	14/0
Organizational position	Administrative and financial	42	11/3
	Hygiene and clinical position	290	78/2
	Deputy headquarters	39	10/5

### Fit index and assessment of the model

We utilized the fitness model to assess the coherence and suitability of the model with the gathered data. We evaluated the adequacy of the conceptual model through two distinct stages, encompassing the model determination segment and the structural component of the model, respectively. During the initial phase, we assessed the model’s reliability and validity.

**Fig. 3:**
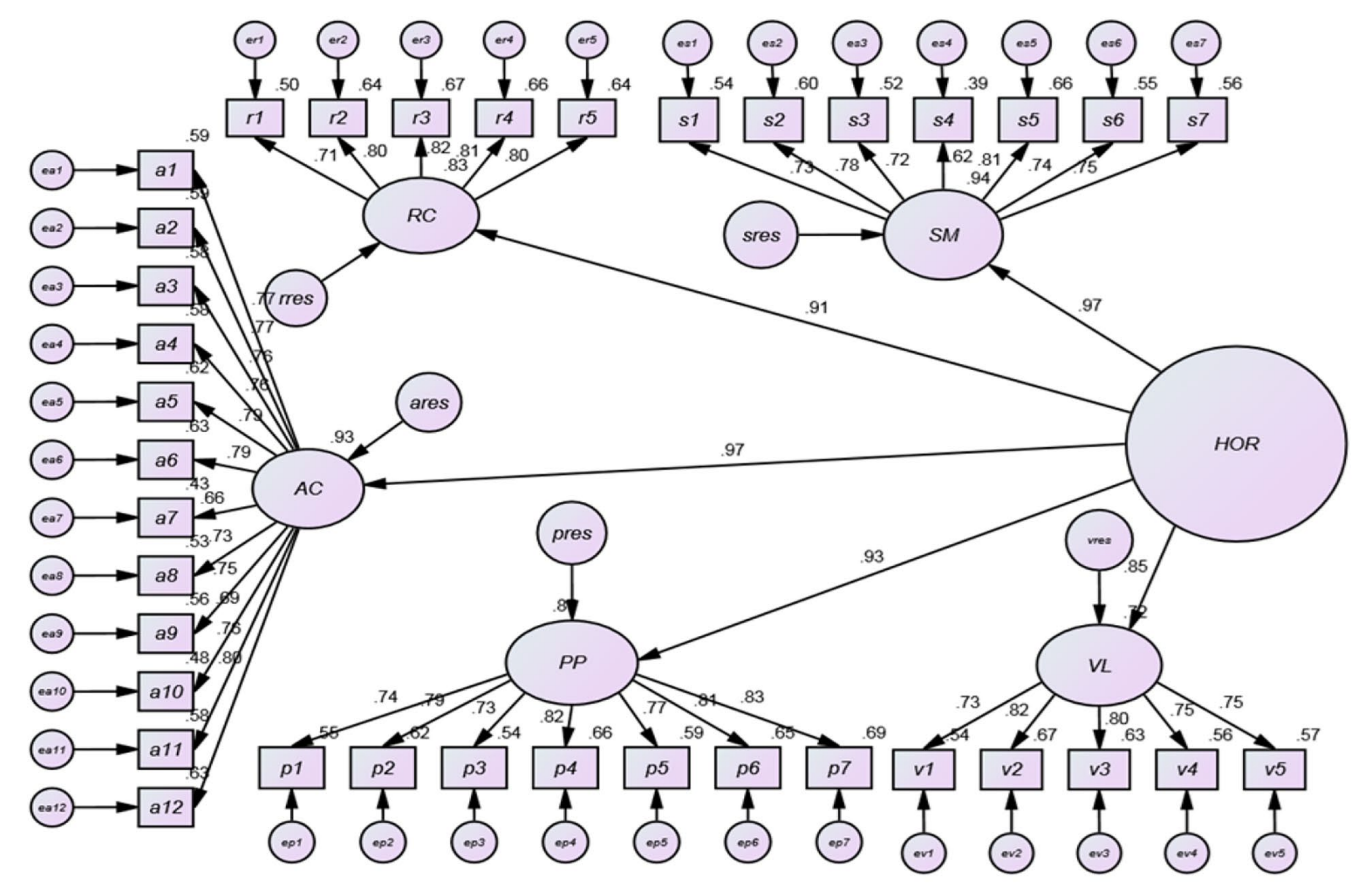
Factors for estimating the standard factor analysis of the primary model

**Fig. 4:**
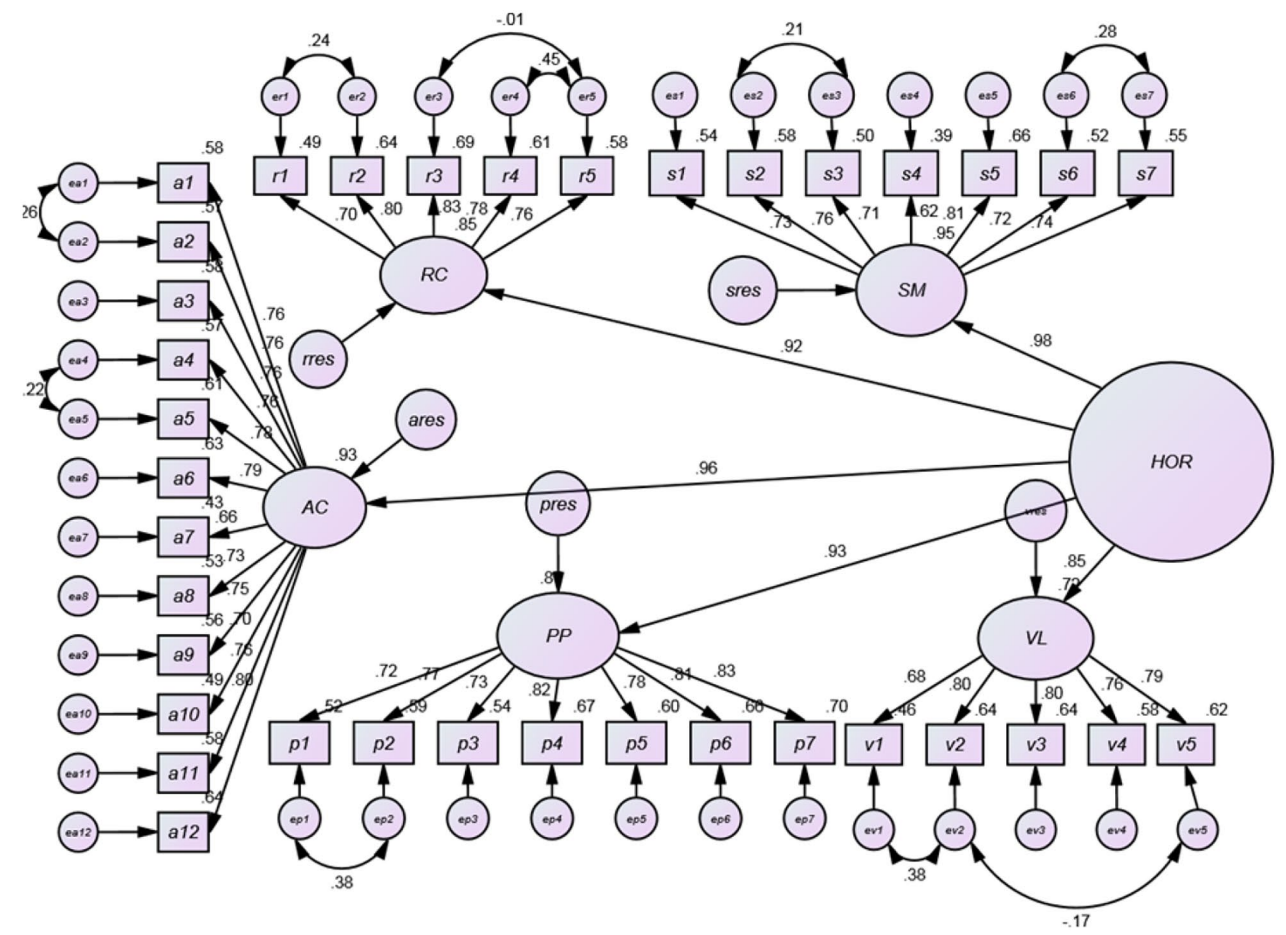
Factors for estimating the standard factor analysis of the proposed model

We conducted a second-order factor analysis to explore the significance of the relationship between organizational resilience and its constituent elements. Based on the results obtained from the standard estimation coefficients of the second-order factor analysis conducted on the hospital’s organizational resilience, all paths were statistically significant at the specified level (Fig. 3). Nevertheless, the computed values for indicators such as GFI and NFI did not fall within the predetermined range, suggesting that the obtained model does not exhibit a sufficiently good fit (Table 2). Consequently, we identified the required modifications to enhance the fit. These adjustments were incorporated into a proposed model, resulting in improved fit indices (Fig. 4).

**Table 2:** Comparison of fitness indices in the primary model and the proposed model

Index	Limit	Proposed model
χ2/df	Less than 3	2/202
GFI	Higher than 0.90	832/0
RMSEA	Less than 0.08	0/057
CFI	Higher than 0.90	0/931
NFI	Higher than 0.90	0/901

### Statistical hypothesis testing

The structural equation modeling analysis revealed a noteworthy correlation between hospital organizational resilience and its associated factors. To assess the significance of the estimated paths, we employed the Student’s T-statistic value for each coefficient, as presented in Table 3. To validate the hypothesis at a 95% confidence level, the t-test value corresponding to the test must be higher than 1.96. As the t-statistic value for all path coefficients surpasses the significance threshold of 1.96, it can be concluded that all path coefficients in the hypothesis are statistically significant. Consequently, all the hypotheses in this research have been confirmed.

**Table 3:** Results of structural equation modeling of dimension in final model

Symbols	Hypotheses			Mean	SD	t statistic	Path coefficient	*P*_value_	Results
H1	Vulnerability	--->	Organizational resilience	3/591	0/812	14/672	0/851	*P* < 0/001	Accepted
H2	Preparedness	--->	Organizational resilience	3/705	0/884	14/546	0/931	*P* < 0/001	Accepted
H3	Support management	--->	Organizational resilience	3/540	0/807	14/708	0/968	*P* < 0/001	Accepted
H4	Responsiveness and adaptability	--->	Organizational resilience	3/748	0/770	-	0/966	-	Accepted
H5	Post- disaster recovery	--->	Organizational resilience	3/657	0/905	15/947	0/909	*P* < 0/001	Accepted

## Discussion

This study aimed to develop a model for assessing the organizational resilience of hospitals in Qazvin province. To achieve this objective, we conducted a comprehensive review and consulted with hospital experts to identify and conceptualize the final model by examining the extracted components. Finally, all experts agreed on five dimensions (vulnerability and preparedness, support management, responsiveness, adaptability during a disaster, and post-disaster recovery) in the form of 36 components. Subsequently, we developed, evaluated, and validated quantitative research instruments. Finally, we constructed the model and made necessary modifications to ensure fit.

The vulnerability dimension, with a path coefficient of 0.851, directly influences approximately 85% of the hospital’s organizational resilience changes. Many studies confirm this finding [4, 24–29]. The components of the vulnerability dimension include identification and analysis of exposure to damage, identification and ranking of structural and non-structural and managerial components of damage, identification of the main clinical and non-clinical processes sensitive to damage, identification, and evaluation of the vulnerable target community, identification and ranking of socio-economic vulnerability components. Regarding this matter, the vulnerability of the hospital is contingent upon its shortcomings and weaknesses during accidents and disasters. Accurate and systematic evaluation of potential risks and vulnerabilities is essential for effective risk planning and management. These studies underscore that a comprehensive and systematic evaluation of a hospital’s vulnerabilities is essential for effective risk planning and management, which enhances the organization’s resilience and ability to withstand and recover from crises.

The planning process should prioritize local risks [30]. In support of this perspective, Carrington et al. emphasize the primacy of comprehending risk and disaster, followed by enhancing disaster risk governance as the second priority. They highlight the importance of conducting risk assessments to identify the hospital’s vulnerability to specific disasters, developing risk management strategies, and enhancing overall disaster resilience on a broader scale [31]. Bazyar et al., in a qualitative study conducted at Ilam University of Medical Sciences, have emphasized the undeniable role of risk information in accurate risk management within the realm of vulnerability [32]. Additionally, Zaboli et al., in their article on elucidating the components of organizational resilience in military hospitals, include vulnerability and safety as the critical dimensions within their model [17]. These studies highlight that effective disaster planning and management should prioritize understanding and assessing local risks and vulnerabilities. Conducting risk assessments to identify specific vulnerabilities of hospitals or healthcare facilities to potential disasters is crucial. This risk information plays an undeniable role in developing accurate risk management strategies and enhancing overall disaster resilience. Vulnerability and safety should be considered critical dimensions in organizational resilience models, particularly in hospitals and military healthcare settings. By prioritizing local risk assessments and incorporating vulnerability considerations, disaster risk governance, and resilience planning can be more effective and tailored to the specific needs and risks of the organization or community.

The preparedness dimension, with a path coefficient of 0.931, directly influences approximately 93% of the hospital’s organizational resilience changes. Many studies confirm this finding [31, 33–37]. The components of the preparedness dimension include creating a rapid warning system and incident command system, developing an operational plan to respond to EOP emergencies, planning for exercises and maneuvers, identifying and analyzing the capacity of equipment, medicine, and physical space, designing a rapid communication system according to the conditions, identifying and analyzing the capacity of human and financial resources, training managers and employees in functional components in critical conditions. In this context, preparedness encompasses anticipating unforeseen events and responding effectively to an accident or disaster. It signifies that the organization is well-positioned to tackle problems by proactively anticipating potential issues, making necessary arrangements, formulating appropriate protocols, identifying and evaluating risks, planning emergency responses, and conducting relevant practical exercises. These measures establish an environment where employees actively engage in safety activities [30]. Supporting this dimension, Mohtadi Ali et al. assert that augmenting staff in the incident command center, sharing planning experiences with the scientific community, and allocating adequate funds have contributed to the hospital’s preparedness [38].

Furthermore, Bazyar et al. highlight that despite the adverse consequences and detrimental impacts on various aspects such as human, economic, social, and environmental domains, disasters are often perceived as an opportunity and a catalyst for development. By capitalizing on limited opportunities, one can be prepared to address the existing disaster and attain a relative readiness to confront future disasters [32]. Additionally, in the study conducted on Indonesian hospitals, Sunindijo et al. affirm that the Hospital Safety Index (HSI) is a valuable tool for assessing the preparedness and resilience of hospitals during emergencies and disasters. It enables the identification of areas that require improvement. The findings can assist hospitals and governmental entities in prioritizing and implementing crucial intervention measures to enhance hospital performance [29]. The studies emphasize that preparedness involves capitalizing on limited opportunities to address existing crises and attain relative readiness to confront future disasters. Tools like the HSI can assist in assessing preparedness and resilience, identifying areas for improvement, and prioritizing interventions to enhance hospital performance during emergencies and disasters. Investing in preparedness measures, including planning, training, resource allocation, and risk assessment, is crucial for hospitals and healthcare organizations to build organizational resilience and effectively respond to emergencies and disasters, ultimately enhancing their overall performance and ability to protect lives and continue operations during critical situations.

The support management dimension, with a path coefficient of 0.968, directly influences approximately 97% of the hospital’s organizational resilience changes. Many studies have pointed to this dimension [17, 31, 38–41]. The components of the support management dimension include setting goals and formulating unit strategies, attracting legal support and developing authority limits, evaluating the logistics situation, evaluating the accreditation standards of disaster and disaster management, monitoring the supply chain and equipping resources, using the creative skills of critical employees, and practical making decision patterns of senior managers in the face of disaster. In this aspect, support management is responsible for supplying resources and essential items to accomplish the operational objectives outlined by the incident command. It involves planning and conducting necessary unit meetings and establishing a support team comprising both internal staff and external reinforcements. These teams are strategically formed to enhance employee engagement and effectively meet increasing demands. As part of support management within an organization, key aspects include defining the responsibilities of each employee, particularly interdisciplinary personnel and new hires, devising strategies for the production and procurement of medicines, medical equipment, and supplies to ensure the provision of healthcare services, and fostering an environment that encourages employees to contribute innovative and practical ideas in response to crises [30]. These studies highlight that effective support management practices, including resource allocation, strategic planning, supply chain management, and fostering a supportive and innovative organizational culture, are essential for hospitals and healthcare organizations to build organizational resilience and respond effectively to emergencies and disasters. By prioritizing and strengthening the support management dimension, hospitals can better align their resources, decision-making processes, and employee engagement to meet the increasing demands and challenges posed by disaster situations.

Bazyar et al. highlight the significance of establishing factories that adhere to the required standards for producing personal protective equipment at national and local levels to support this dimension. This is crucial because the availability of such factories at the national and provincial levels was limited, resulting in significant challenges for medical and healthcare personnel in protecting themselves. Additionally, leveraging the capacity of health donors during disaster events proved to be a valuable resource in the province [32].

The dimension of responsiveness and adaptability during a disaster, with a path coefficient of 0.966, directly influences approximately 96% of the organizational resilience changes within the hospital. Many studies have pointed to this dimension [28, 31, 32, 41–43]. The components of responsiveness and adaptability during a disaster include taking measures to continue the vital functions of the hospital, managing the capacity of diagnostic and preclinical services according to the needs, the feasibility of increasing the capacity of the physical space suitable for emergency evacuation, managing the waiting time of patients to receive services based on prioritization in triage, strengthening the mechanisms of referral and emergency dispatch of patients, optimal management of energy supply, storage and consumption, collection and documentation of all information related to the accident, optimal use of the total capacity of beds and medical equipment in all departments, monitoring and controlling the correct triage process of patients, signing a memorandum of understanding to increase the capacity of waste management, laundry and cold storage, calling and dispatching quick response teams in emergencies. Concerning this matter, the response phase is when the incident response plan is implemented, aiming to save human lives, provide immediate medical assistance, mitigate and repair damages to existing systems, and deliver necessary services to the affected individuals. During a disaster, key factors include predictive capability, identification of significant environmental changes, adherence to compliance through continuous performance monitoring, and the system’s ability to adapt gradually or entirely in response to the evolving environment. These measures enhance the hospital’s critical performance by increasing its agility and responsiveness [30]. These studies show that the support management dimension influences a hospital’s organizational resilience and ability to respond effectively to emergencies and disasters.

Corroborating this dimension, Shirali et al., in their study investigating eight hospitals in the southwestern region of Iran, highlight that these hospitals face challenges in the response phase. The reasons behind these challenges include insufficiently trained personnel, limited resources, and equipment, inadequate emergency operation activation, deficient planning for shelter and collective care, as well as insufficient availability of blood bank, water, food, pharmacy, and inadequate space for admitting and treating the injured [33].

Furthermore, Blanchet et al. explore the resilience of a health system and emphasize that adaptability is the ability of system actors to respond to stresses and shocks effectively [24]. Additionally, Sunindijo et al. assert that the hospital’s response during a disaster encompasses various factors. These factors include expanding available space to accommodate mass disasters, ensuring sufficient logistic equipment for significant disasters, implementing effective methods for patient transfer and acceptance during emergencies and disasters, having a plan for infection prevention and control, and employing protocols for managing deceased individuals [29]. These studies highlight that while responsiveness and adaptability are crucial, hospitals and healthcare organizations must address the existing challenges and gaps in their preparedness and response capabilities. This may involve investing in personnel training, resource allocation, emergency operation plan development and activation, contingency planning for shelter and care, ensuring adequate supplies and space, and establishing protocols for various aspects of disaster response, such as patient transfer, infection control, and management of deceased individuals.

The post-disaster recovery dimension, with a path coefficient of 0.909, directly influences approximately 90% of the changes in organizational resilience within the hospital. Many studies have pointed to this dimension [17, 26, 32, 38, 44, 45]. The components of this dimension include the compilation of the final assessment report of damages and costs, the compilation of databases of experiences gained for learning, databases of experiences gained for lessons, the analysis of the physical, mental, and social health status of employees involved in the disaster. They are adopting stabilization strategies and increasing the motivation of active members in disaster management. The recovery phase encompasses all actions undertaken to stabilize and restore the hospital to its pre-incident state. This process involves the reconstruction of damaged buildings and structures, restoration of essential infrastructures, community resettlement efforts, and providing necessary mental health services for the survivors [30]. Supporting this aspect, Carrington et al. emphasize the importance of enhancing disaster preparedness for achieving effective response and emphasizing “better reconstruction” during recovery, rehabilitation, and reconstruction. This aspect is identified as the fourth priority in their study [31].

Furthermore, in a review study conducted by Fallah Ali Abdi et al., it is mentioned that the indicators associated with recovery and response are regarded as organizational agility, which is a resilience measure reflecting the hospital system’s ability to prioritize and restore performance swiftly, while also preventing future disruptions [26]. Additionally, Shir Ali et al. highlight that these hospitals face unsatisfactory conditions regarding post-disaster recovery. The analysis of the findings revealed significant deficiencies, such as the absence of a disaster recovery plan and ineffective recovery management practices [33]. These findings highlight the importance of conducting comprehensive assessments, documenting experiences and lessons learned, addressing employees’ well-being in the disaster, and implementing strategies to stabilize and motivate the workforce during the recovery phase.

### Limitations

This is the first kind of study conducted in Iran using a mixed-method design. However, we had some limitations: (1) the absence of studies utilizing the modeling method to extract components from the literature review. However, an attempt was made to partially compensate for this limitation by employing a robust search strategy and extracting various components from the available studies. (2) Our study was restricted focus on the panel of experts from Qazvin City. It is recommended that future research endeavors involve expert panels from diverse specialties in various provinces across the country to enhance the conceptualization process.

## Conclusion

Enhancing organizational resilience is crucial for hospitals to improve preparedness and minimize casualties during crises and disasters. Developing localized tools to measure this resilience can serve as a foundation for strengthening hospitals’ responsiveness during such events. As resilience is a relatively new concept in disaster literature, it provides an opportunity for managers and policymakers to evaluate hospitals’ resilience levels, identify vulnerabilities, assess service continuity during crises, and implement effective rehabilitation programs. The COVID-19 pandemic has underscored the critical need for measuring resilience. The research findings indicate that a model encompassing vulnerability, preparedness, support management, responsiveness, adaptability during disasters, and post-disaster recovery is suitable for assessing hospital resilience in crises and disasters. Factors such as human resource scarcity, hospital specialization, and limited trauma and burn center capacity should be considered. During the vulnerability stage, efficient information and resources are crucial for identifying and assessing hospital exposure to various crises. Enhancing information technology infrastructure and conducting training courses are advisable for effective communication and preparedness. Establishing memorandums of understanding with relevant organizations can improve waste management, landfill capacity, and cold storage capabilities. Ultimately, a proactive and flexible organizational framework should be in place to prepare units, facilities, and human resources based on the research model’s components. Further studies could investigate the effects of a hospital’s economic problems on its resilience, hospital staff’s participation in coping with incidents and disasters, and its effects on enhancing hospital resilience, as well as examine the social and cultural impacts on hospital resilience. Additionally, research on the effect of various interventions on the development and improvement of hospital resilience in incidents and disasters would be valuable. Moreover, a study presenting an organizational resilience model in other sectors could provide insights for improving resilience across the healthcare system.

### Electronic supplementary material

Below is the link to the electronic supplementary material.

Supplementary Material 1

## Data Availability

The datasets used and/or analyzed during the current study available from the corresponding author on reasonable request. The entire dataset is in Farsi language. The Data can be available in English language for the readers and make available from the corresponding author on reasonable request.

## Abbreviations

HOR: Organizational resilience of the hospital
VL: vulnerability
PP: preparedness
AC: answer crisis (responsiveness and adaptability)
SM: Support management
RC: Recovery after the crisis
