# Supplementary material for: A model of the organizational resilience of hospitals in emergencies and disasters
Source: BMC Emerg Med. 2024 Jun 24;24:105. doi: 10.1186/s12873-024-01026-6 (PMC11197230; doi:10.1186/s12873-024-01026-6)
Supplement: Supplementary file 1 — Supplementary Material 1 [file 12873_2024_1026_MOESM1_ESM.pdf]

"Effective factors on organizational resilience in crises and disasters".

Dear Sir/Madam

This questionnaire evaluates the importance of " Effective factors on the organizational resilience in crises and disasters" in two parts of demographic information and information related to the analytical goals of the research. The following questionnaire contains 5 dimensions and 36 components. Please read all the questions carefully and express your opinion regarding the importance of each component as very low, low, medium, high and very high. Thank you in advance for your kind attention.

Thanks

Research team

First part) Write your demographic information:

1) Age: .... years

2) Gender: Male□ Female□

3) Level of education: Less than bachelor's degree□ Bachelor's degree□ Master's degree□ PhD□ MD□

4) Service history: less than 10 years□ 10-20 years□ more than 20 years□

5) Organizational post: Administrative-financial centers□ Health centers□ University headquarters-deputies and research centers□

Second part) Answer the following questions according to their importance from very low, low, medium, high and very high:

Row	Title	Importance				
		very low	low	medium	high	very high
**Vulnerability components**						
1	Identification and analysis of exposure to damage					
2	Identification and ranking of structural and non-structural harmful components, management					
3	Identification of major clinical and non-clinical processes sensitive to injury					
4	Identification and evaluation of the vulnerable target community					
5	Identifying and ranking the components of socio-economic vulnerability					
**Preparedness components**						
6	Creating a quick warning system and an incident command system					
7	Development of an operational response plan in emergencies (EOP)					
8	Planning for training and maneuvers					
9	Identifying and analyzing the capacity of equipment, medicine and physical space					
10	Designing a fast communication system according to the conditions, between decision-makers and executive agents					
11	Identifying and analyzing the capacity of human and financial resources					

12	Training managers and employees' functional components in critical situations					
**Support management components**						
13	Goal-setting and formulation of unit strategies					
14	Attracting legal protections and developing authority limits					
15	Assessment of the logistics situation					
16	Evaluation of accreditation standards for disaster management					
17	Monitoring the supply chain and equipping resources					
18	Utilizing the creative skills of key employees					
19	Applying the decision-making patterns of senior managers in a crisis					
**Components of response and adaptability during crisis**						
20	Taking measures to continue the vital functions of the hospital					
21	Managing the capacity of diagnostic and paraclinical services					
22	Feasibility of increasing the capacity of physical space for emergency evacuation					
23	Management of patients waiting time to receive services based on prioritization in triage					
24	Strengthening mechanisms for referral and emergency dispatch of patients					
25	Optimal management of energy supply, storage and consumption					

26	Collecting and documenting all information related to the incident					
27	Optimal use of the full capacity of beds and medical equipment in all departments					
28	Monitoring and controlling the patients' right triage process					
29	Signing a memorandum of understanding to increase the capacity of waste management, laundry and cold storage					
30	Calling and dispatching rapid response teams in emergencies					
31	Implementation of congestion management process					
**Components of post-crisis recovery**						
32	Compilation of the final evaluation report of damages and costs					
33	Compilation of databases of acquired experiences for learning					
34	Developing a strategy for redesigning and rebuilding the physical structure to prevent and reduce the effect of future risks					
35	Analysis of the physical, mental and social health status of employees involved in the crisis					
36	Adopting stabilization strategies and increasing the motivation of active members in crisis management					
